# Reversal of epigenetic silencing of MHC class I chain-related protein A and B improves immune recognition of Merkel cell carcinoma

**DOI:** 10.1038/srep21678

**Published:** 2016-02-23

**Authors:** Cathrin Ritter, Kaiji Fan, Kelly G. Paulson, Paul Nghiem, David Schrama, Jürgen C. Becker

**Affiliations:** 1 Translational Skin Cancer Research, German Cancer Consortium (DKTK), University Hospital Essen, Germany; 2 German Cancer Research Center (DKFZ), Heidelberg, Germany; 3 Department of Dermatology, Medical University of Graz, Graz, Austria; 4 Division of Dermatology and Medical Oncology, Department of Internal Medicine, University of Washington, Seattle, WA; 5 Department of Dermatology, University Hospital Würzburg, Würzburg, Germany

## Abstract

Merkel cell carcinoma (MCC) is a virally associated cancer characterized by its aggressive behavior and strong immunogenicity. Both viral infection and malignant transformation induce expression of MHC class I chain-related protein (MIC) A and B, which signal stress to cells of the immune system via Natural Killer group 2D (NKG2D) resulting in elimination of target cells. However, despite transformation and the continued presence of virally-encoded proteins, MICs are only expressed in a minority of MCC tumors *in situ* and are completely absent on MCC cell lines *in vitro*. This lack of MIC expression was due to epigenetic silencing via MIC promoter hypo-acetylation; indeed, MIC expression was re-induced by pharmacological inhibition of histone deacetylases (HDACs) both *in vitro* and *in vivo*. This re-induction of MICs rendered MCC cells more sensitive to immune-mediated lysis. Thus, epigenetic silencing of MICs is an important immune escape mechanism of MCCs.

Merkel cell carcinoma (MCC) is an aggressive skin cancer with neuroendocrine features. The histogenesis of MCC remains controversial, with either epidermal stem cells or pre/pro-B cells as possible cells of origin^1^. Currently, there are no approved therapies for advanced MCC that is not surgically resectable, thus almost half of the patients diagnosed with MCC will die from the disease^2^. This situation is compounded because the reported incidence of MCC has more than tripled over the last few decades^3^. This increase in incidence is true across all age groups, indicating that the aging general population is not the sole reason for the increased incidence. Nevertheless, advanced age is still one of the relevant risk factors among UV exposure and immune suppression^4^.

The strong correlation of MCC and immune suppression prompted the discovery of a polyomavirus associated with MCC, termed Merkel cell polyomavirus (MCPyV)^5^. MCPyV is present and clonally integrated in at least 80% of MCCs^6^. Most MCPyV positive MCC cell lines critically depend on virally encoded transforming early genes, i.e. small and large T-antigen, in order to maintain the oncogenic phenotype^7^,^8^. The continuous expression of these viral proteins helps explain the exquisite immunogenicity of MCC. Notably, despite the highly aggressive phenotype of MCC, spontaneous regression or regression after cessation of immune suppressive measures are well established^9^. Moreover, we and others have recently demonstrated the presence of spontaneous adaptive cellular immune responses specific for epitopes derived from the MCPyV early genes in peripheral blood of MCC patients^10^,^11^. However, despite the continuous expression of the relevant antigens and the presence of respective specific cytotoxic T-cell responses, it is obvious that clinically manifest MCCs are able to escape destruction by the immune system. While this fact can be readily explained by a general immune suppression in approximately 10% of the patients^12^, the immune escape mechanisms of MCC are less clear for the remaining 90%^13^.

Natural Killer group 2D (NKG2D) is a lectin-like type 2 transmembrane receptor encoded by the gene *Klrk1* (killer cell lectin-like receptor subfamily member 1) and is part of a critical pathway signaling cellular stresses to the innate and adaptive immune system. Charged residues in the transmembrane region enable NKG2D to pair with the signaling adaptor protein DAP10, which is essential for NKG2D surface expression and downstream signaling to PI3K and GRB2^14^. These signaling molecules then stimulate proliferation, cytokine production, immune cell activation, and cytotoxic potential of NK and T cells^14^. A recent study suggested a link between the Natural Killer group 2D (NKG2D) receptor system and up-regulation of immune responses to MCC. Specifically, transcriptional analyses of MCC tumors revealed that NKG2D was among the highest expressed mRNAs in tumors obtained from patients with a good prognosis^15^. However, these tumors represented a minority of patients, suggesting most MCCs evade NKG2D signaling as a means of immune escape.

The NKG2D ligands include UL16-binding proteins (ULBPs) as well as the MHC class I chain-related protein (MIC) A and B family^16^. MICA and MICB are present at low to undetectable levels in normal cells, but are induced by cellular stresses including infectious agents and neoplastic transformation. Indeed, MICA and MICB are highly expressed in a number of solid tumors like carcinomas of the breast, colon, kidney, ovary, or prostate^17^, as well as in melanoma^18^. However, NKG2D expression renders tumor cells more susceptible to elimination by the immune system^19^. The importance of MICA and MICB induced NKG2D-signaling for immune surveillance of virally infected and transformed cells is highlighted by the fact that viruses and cancer cells have developed mechanisms to interfere with this interaction^14^. These mechanisms include shedding of surface expressed molecules, binding and retaining of MICA and MICB proteins in the cytoplasm, over-expression of *MICA* and *MICB* mRNA-targeting microRNAs, as well as other epigenetic mechanisms such as chromatin remodeling^14^,^20^.

Viral carcinogenesis should predispose MCC for induction of MICA and MICB expression; however, when screening for the respective mRNA expression using publicly available data from the Gene Expression Omnibus (GEO), we observed that both *MICA* and *MICB* mRNA were rarely present in MCC. Prompted by this observation, in the present study we confirmed these data in an independent set, and extended this notion to the protein level. Furthermore, we demonstrate that this lack of MICA and MICB expression in MCC is due to epigenetic silencing by promoter hypo-acetylation. Notably, MICA and MICB expression, particularly MICB expression, can be induced by histone deacetylase inhibitors, which in turn rendered the MCC cells more susceptible to lysis by cytotoxic lymphocytes. These findings open new avenues for therapy of advanced MCC particularly in combination with immune modulating molecules such as immune checkpoint blocking antibodies.

## Results

### MCC tumors express low levels of *MICA* and *MICB* mRNAs

Since both viral infection and malignant transformation induce expression of the immune activating NKG2D ligands MICA and MICB, we screened for the respective mRNA expression among 75 MCC tumors from 61 patients and a number of MCC cell lines. For this, we employed 2 publicly available gene expression arrays obtained online from GEO (accession numbers GSE22396^15^ and GSE 39612^21^; Supplementary Fig. 1). Somewhat unexpectedly, *MICA* mRNA was expressed only at very low levels in the MCC tumors and cell lines when compared to genes commonly expressed in MCCs such as *RB1*, *E2F2*, *ENO2* or *RPLP0*. The *MICB* mRNA expression level was also low compared to those genes, but generally higher than for *MICA*. On the GSE22396 Array a subset of tumors (24%) were characterized by moderate to high levels of *MICB* mRNA. Notably, patients with higher levels of *MICB* mRNA in their tumors where characterized by better outcomes. In line with this, in MCPyV positive tumors, *MICB* mRNA expression correlated with the gene expression signature for infiltrating immune competent cells, a feature that had been associated with a good prognosis previously^15^ (Supplementary Fig. 2).

### MCC tumors largely lack MICA and MICB expression *in situ*

**Figure 1:**
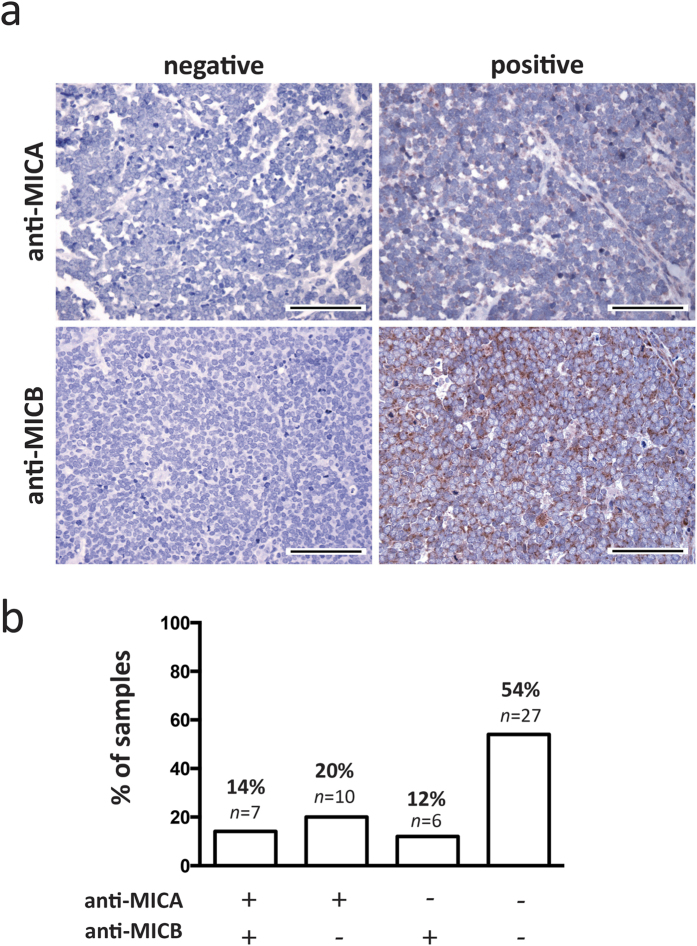
MICA and MICB expression of MCC tumors *in situ*. MCC tumor samples from 34 patients were analyzed by immunohistochemistry for expression of MICA and MICB. (**a**) Samples were classified as positive or negative. Representative negative and positive samples are depicted at 40× magnification, scale bars are 100 μm. The positive examples represent the strongest obtained signal with the respective antibody. (**b**) Tumor samples were stratified into 4 groups: Double positive for MICA and MICB (+/+, 14%, n = 7), only positive for MICA (+/−, 20%, n = 10), only positive for MICB (−/+, 12%, n = 6), or double negative (−/−, 54%, n = 27).

Next, we analyzed tissue microarrays (TMA) comprising 134 FFPE fixed paraffin embedded MCC tumors of 99 patients by immunohistochemistry (IHC) using an antibody reacting with both MICA and MICB to determine MICA/B protein expression^18^ (Fig. 1a). MICA/B protein was present in only 18% of MCC tumors, while 82% were negative (Supplementary Fig. 3).

The samples used in the cDNA microarray and the TMA were partly overlapping. Thus, to confirm the lack of MICA and MICB expression in a completely independent set of samples, we analyzed 50 additional MCC tumor samples of 34 patients for their MICA and MICB expression by IHC in conventional tumor sections. The use of sections of the whole tumor also allowed us to determine the expression pattern of MICA and MICB. A heterogeneous expression pattern of other immune modulatory molecules such as CD274 (*aka* PD-L1) had recently been described for MCC^22^.

Tumors classified as negative or positive for MICA and MICB expression are exemplarily depicted in Fig. 1a; notably, the strongest staining observed for the respective antibody is depicted. In line with higher *MICB* mRNA expression, MICB staining intensity was stronger than that of MICA. Overall we observed that more than half (54%) of the lesions expressed neither MICA nor MICB protein, 20% were weakly positive for only MICA, and 12% were classified positive for only MICB; and only 14% of the tumors stained positive for both MICA and MICB (Fig. 1b). The effective frequency of MICA and MICB expression remained essentially the same when calculated for each patient instead of the individual lesions (Supplementary Fig. 4). Indeed, when examining multiple lesions, i.e. primary tumors and metastatic lesions obtained from the same patients, the expression pattern of the MICA and MICB was concordant (data not shown). Similarly, with respect to a possible heterogeneity of MICA and MICB expression within the tumor, our analyses revealed that both the expression as well as the lack of it was homogenous throughout the tumor. This homogenous intralesional and intraindividual expression pattern suggests a rather robust mechanism for suppressed protein expression, such as epigenetic silencing.

### MICA and MICB expression in MCC cell lines

**Figure 2:**
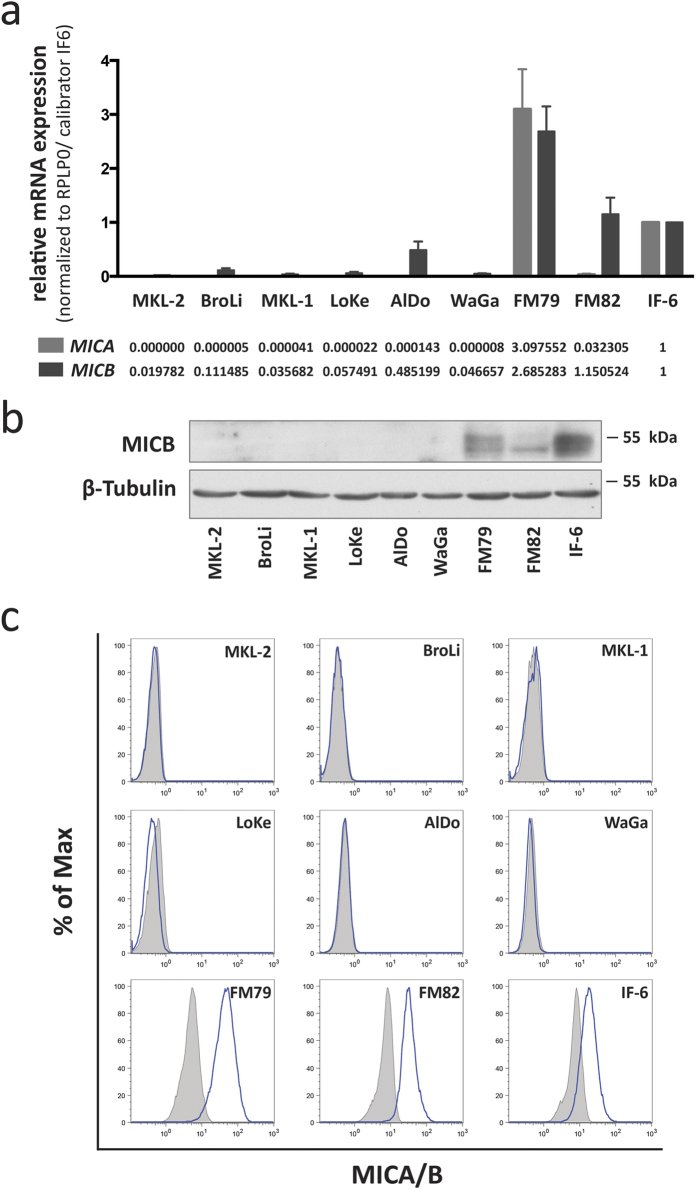
MCC cell lines do not express MICA and MICB protein despite low levels of *MICA* and *MICB* mRNA. (**a**) MICA (light grey) and MICB (dark grey) mRNA expression was determined by qRT-PCR in MCC cells. Relative expression levels were calculated by normalization of C_T_ values to RPLP0 and calibration to the melanoma cell line IF6. (**b**) MICB protein expression of whole cell lysates was determined by immunoblot; β-tubulin served as a loading control. (**c**) MICA/B cell surface expression was determined by flow cytometry using an antibody recognizing both MICA and MICB (clone 6D4; blue line); matched isotype control is depicted as grey filled area. Melanoma cell lines FM79, FM82 and IF6, served as positive control for MICA and MICB expression in all assays illustrated in this figure.

To explore the underlying mechanism, we determined whether the suppression of MICA and MICB expression was robust enough to be maintained when cells were propagated *in vitro*, in cell culture conditions. This question is of particular relevance since *in vitro* culture of cells is a well-established factor known to induce MICA and MICB expression^23^,^24^. We therefore performed qRT-PCR with *MICA* and *MICB* mRNA specific primers using the melanoma cells FM79, FM82 and IF6 as positive controls (Fig. 2a). These analyzes demonstrated that *MICA* mRNA in MCC cell lines was only expressed in very low amounts, i.e. close to or below the detection threshold; indeed, relative expression values calibrated to IF6 ranged close to zero. Similar to the results from the analyses of the MCC tumor samples, *MICB* mRNA expression was low, but still higher than *MICA* mRNA, with relative expression ranging from approximately 0.02 to 0.48 when calibrated to the positive control melanoma cell line IF6’s mRNA expression. Inconsistencies between mRNA and protein expression for MICB have been repeatedly described^25^, thus, to determine whether the detected *MICB* mRNA was indeed translated into protein we performed immunoblots of total cell lysates demonstrating that MICB protein was below the detection limit even for the AlDo MCC cell line characterized by the highest *MICB* mRNA expression (Fig. 2b). Accordingly, flow cytometric analysis for MICA/B membrane expression demonstrated no expression in any of the analyzed MCC cell lines (Fig. 2c). To determine how broadly NKG2D mRNA expression was suppressed, we subjected MCC cells to a number of stress factors known to induce NKG2D ligand expression^24^,^26^. These stressors included serum starvation, high concentrations of DMSO, heat shock at 41.5 °C, 1000 U/ml interferon α, and 1000 U/ml interferon γ. These interventions had no or only negligible effects on MICA/B surface expression (Supplementary Fig. 5a–f). Finally, we took advantage of a recently established inducible knock down system for viral T-antigens to stress the cells by withdrawal of the oncogene to which they are addicted; this knock down had no effect on MICB expression (Supplementary Fig. 5g).

The complete lack of MICA and MICB protein expression in MCC cell lines even under diverse cell stress conditions suggests that MICA and MICB silencing in MCC is an active and robust process.

### *MICA* and *MICB* promoters are silenced by histone hypo-acetylation

**Figure 3:**
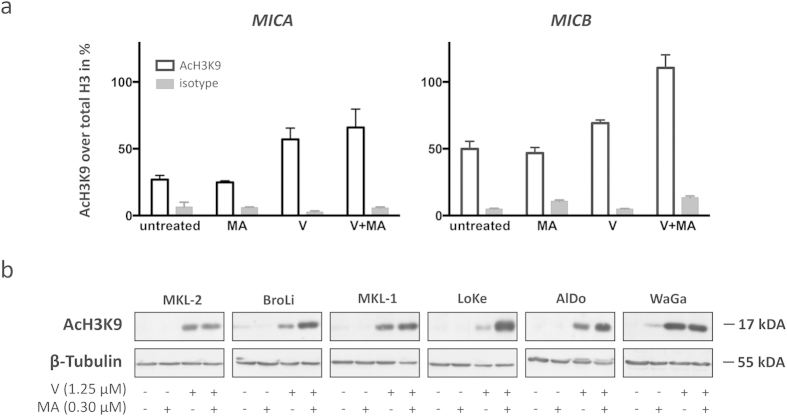
Vorinostat alone or in combination with mithramycin A increases global as well as *MICA* and *MICB* promoter-specific histone H3 Lysine 9 (H3K9) acetylation in MCC cell lines. (**a**) Chromatin immunoprecipitation (ChIP) assay was performed with untreated, vorinostat (V), mithramycin A (MA), or the combination thereof (V+MA) treated WaGa cells followed by a qRT-PCR using MICA or MICB promoter specific primers. C_T_ values of anti-acetyl-H3K9 (AcH3K9) antibody or rabbit IgG isotype control precipitated DNA were normalized to total histone H3 antibody as described in materials and methods. White bars represent the percentage of acetylated H3K9, grey bars the respective control. Experiments were performed in duplicates and results are expressed as mean ± SEM. (**b**) Global H3K9 acetylation of untreated, V, MA, or V+MA treated MCC cell lines was determined by immunoblot with the same AcH3K9 antibody used in the ChIP assay; β-tubulin served as loading control.

The robust silencing of MICA and MICB expression in MCC observed *in vivo* and *in vitro* suggests that this regulation takes place on a transcriptional or epigenetic level rather than post-transcriptionally^27^. We therefore determined histone H3K9 acetylation at the *MICA* and *MICB* promoter region of WaGa cells as a known indicator for transcriptional activity. Chromatin immune precipitation (ChIP) assays clearly demonstrated histone hypo-acetylation at both the *MICA* and *MICB* promoter: Only ~20% of histones in the MICA promoter and ~50% of histones in the *MICB* promoter were acetylated in WaGa cells (Fig. 3a). The lower acetylation levels of histone H3 at the MICA promoter compared to the *MICB* promoter in WaGa cells is in accordance with a lower expression of MICA compared to *MICB* mRNA as described above.

### *MICA* and *MICB* promoter acetylation is inducible

Apparently, the expression of MICA and MICB in MCC cell lines is silenced via histone H3 hypo-acetylation at their promoter region. To test whether the silencing of these NKG2D ligands is indeed due to histone hypo-acetylation or if additional mechanisms for MICA and MICB suppression are operative^14^, we next tested if their expression could be induced by reversal of histone hypo-acetylation. WaGa cells were subjected to a clinically relevant concentration of 1.25 μM (the plasma concentration reached by currently applied treatment regimens) of the histone deacetylase (HDAC) inhibitor vorinostat (SAHA, Zolinza)^28^. When WaGa cells were cultured for 24 hours in the presence of vorinostat, there was an induction of histone acetylation at the *MICA* and *MICB* promoter regions (Fig. 3a). However, with ~56% of histone acetylation in the MICA and ~70% of histone acetylation in the *MICB* promoter, this induction was rather modest. We therefore combined vorinostat with mithramycin A, a drug that has synergistic effects with HDAC inhibitors by (i) transcriptionally inhibiting the compensatory inductions of certain HDACs^29^ and (ii) by preventing the formation of SP1/HDAC inhibitory complexes at the promoters’ GC box^30^. We confirmed these synergistic effects in the MCC cell lines: Treatment with mithramycin A alone already reduced transcription of most class I and II HDAC genes in MCC cell lines and, most importantly, mithramycin A prevented the regulatory induction of HDACs by vorinostat (Supplementary Fig. 6). Subsequently, ChIP assays of accordingly treated MCC cells revealed that the addition of mithramycin A boosted vorinostat induced histone acetylation resulting in an acetylation of ~66% of histones at the MICA promoter and almost complete histone acetylation at the *MICB* promoter. Treatment with mithramycin A alone had negligible effects on histone acetylation levels in the *MICA* and *MICB* promoter of WaGa cells.

To extend this observation to a larger series of MCC cell lines, we performed an immunoblot for global histone acetylation using the same anti-AcH3K9 antibody we employed for the ChIP assay. This analysis confirmed the increased acetylation of global histones upon combined vorinostat and mithramycin A treatment in 4 out of 6 cell lines (Fig. 3b). In MKL-2 and in WaGa cells we did not observe a further increase in global histone H3 acetylation by adding mithramycin A. This observation was unanticipated, since we observed a synergistic effect of the combined drugs on histone H3 acetylation at the *MICA* and *MICB* promoter regions of WaGa cells (Fig. 3a). A possible explanation is that both the *MICA* and *MICB* promoter regions may be more sensitive to mithramycin A induced histone acetylation due to the presence of a SP1 binding site (GC Box)^30^.

### MICA/B expression can be re-induced on MCC cells *in vitro*

**Figure 4:**
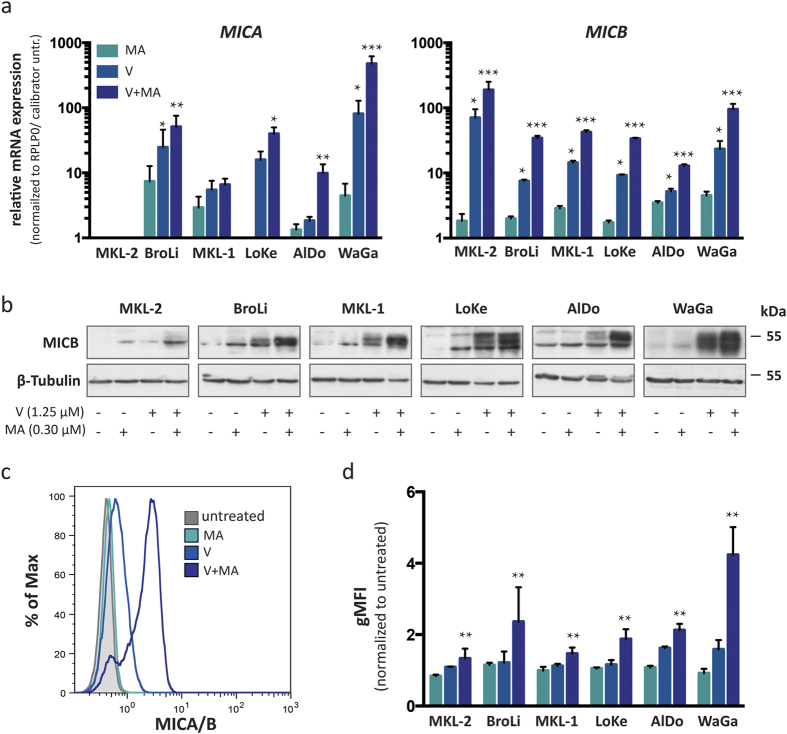
Induction of MICA and MICB expression by vorinostat alone or in combination with mithramycin A. MICA and *MICB* mRNA and protein expression of untreated MCC cell lines were compared to the respective expression after treatment with vorinostat (V, light blue), mithramycin A (MA, turquoise), or the combination thereof (V+MA, dark blue). (**a**) mRNA expression of MICA and MICB was determined by qRT-PCR in duplicates in three independent experiments; C_T_ values were normalized to RPLP0 and calibrated to the ΔC_T_ value of the respective untreated cell line; relative mRNA expression is depicted on a logarithmic scale (log 10) ± SEM. (**b**) MICB expression in whole cell lysates of MCC cell lines was detected by immunoblot using a MICB specific antibody; β-tubulin served as loading control. (**c,d**) MICA/B cell surface expression was determined by flow cytometry using an antibody recognizing both MICA and MICB (clone 6D4), which is exemplified for WaGa (**c**); the results for all cell lines are depicted as the geometric mean fluorescence intensity (gMFI) of MICA/B staining, normalized to the respective untreated cell lines ± SEM in three independent experiments (**d**). Statistical analysis was performed using the Friedman test as indicated.

Since the combined treatment of MCC cells with vorinostat and mithramycin A induced strong histone acetylation at the *MICA* and *MICB* promoter, we next tested whether this also leads to an increased transcription of MICA and MICB genes. First, we determined the respective mRNA expression with or without treatment by quantitative RT-PCR (Fig. 4a). The combination of vorinostat and mithramycin A led to increased *MICA* and *MICB* mRNA expression in all tested MCC cell lines, with the exception of MKL-2 in which no *MICA* mRNA was detected. Notably, the synergistic effects of vorinostat and mithramycin A were so strong that the relative expression to the respective untreated cell line in Fig. 4a is depicted on a logarithmic (log10) scale. Next, immunoblot and flow cytometry assays confirmed that the increased mRNA expression translated into MICB protein expression in general (Fig. 4b) and more importantly membrane expression (Fig. 4c,d). The degree of induction of membrane bound MICA/B was comparable to that of total MICB protein expression, suggesting that most of the induced MICA/B proteins are transported to the cell surface and no additional post-translational mechanisms interfere with MICA/B surface expression in MCC cells. It should be noted, however, that the combined treatment did not affect all cells of the individual cell lines to the same extent. For a small but distinct subpopulation encompassing between 10 to 15% of the total population, MICA/B surface expression was almost unaltered by this treatment (Fig. 4c).

When we subjected MCC cells to increasing concentrations of vorinostat starting at 1.25 μM used throughout the previous experiments to 10 μM we observed that vorinostat alone is capable of inducing strong MICA/B surface expression in the majority of AlDo, BroLi and WaGa cells if present at high concentrations, whereas in LoKe, MKL-1 and MKL-2 cells the magnitude of vorinostat inducible MICA/B expression is lower and the plateau of expression is reached already at intermediate concentrations (Supplementary Fig. 7). This observation together with the observation that a subpopulation of cells did not respond to the synergistic effect of mithramycin A suggests that histone hypo-acetylation at the *MICA* and *MICB* promoter is maintained by different mechanisms depending on both the cell line per se as well as the functional state of the cell.

An alternative HDAC inhibitor, i.e. the “classical” HDAC inhibitor trichostatin A (TSA), alone or in combination with mithramycin A produced essentially the same results as observed with the “second-generation” HDAC inhibitor vorinostat suggesting that only class I and II HDACs are involved in silencing MICA and MICB (Supplementary Fig. 8).

### Induction of MICA/B enhances LAK cell mediated lysis of MCC cells

**Figure 5:**
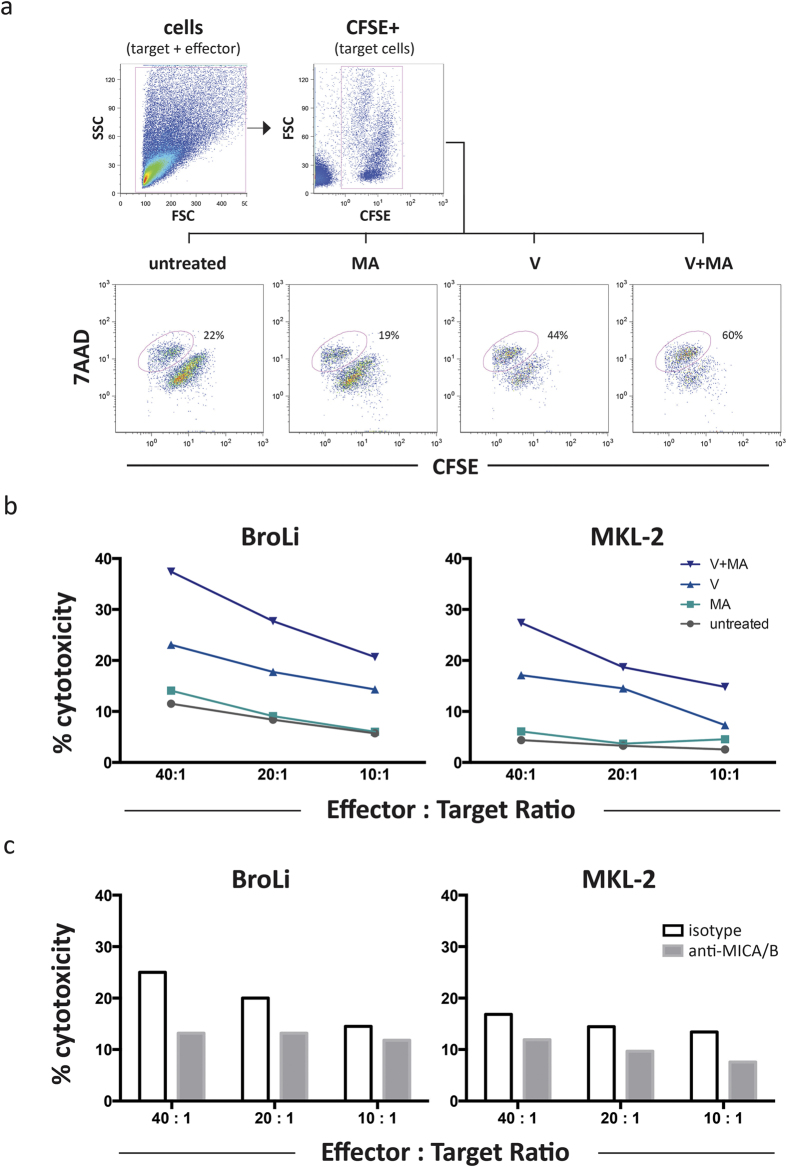
Inhibition of HDACs in MCC cell lines increased their susceptibility to LAK cell mediated lysis, which is is subdued by MICA/B blockade. The flow cytometry based cytotoxicity assay was performed as described in Material and Methods. (**a**) The gating strategy is illustrated for untreated and treated BroLi cells used at an effector to target ratio of 40:1; target cells were gated as CFSE positive cells in an FSC/CFSE plot, lysed target cells were defined as 7AAD/CFSE double positive cells and are quantified as percentage of all target cells. (**b**) Untreated (grey), vorinostat (V, light blue), mithramycin A (MA, turquoise), or the combination thereof (V+MA, dark blue) treated BroLi and MKL-2 cells served as target cells for LAK cells at the indicated effector to target ratios in a 4h cytotoxicity assay. The lysis of the respective target is given as average of three independent experiments. (**c**) Vorinostat plus mithramycin A treated BroLi and MKL-2 cells served as target cells for LAK cells at the indicated effector to target ratios in a 4h cytotoxicity assay in the presence of saturating amounts of a MICA/B specific blocking antibody (grey bars) or an isotype control antibody (white bars); Fc receptors of effector cells were blocked by saturating amounts of F(ab)2 fragments.

The expression of NKG2D ligands such as MICA and MICB render tumor cells more susceptible to being killed by NK and T cells. Notably, T-cell activation can be mediated by NKG2D even without contribution of TCR-recognition^31^. Thus, we addressed whether induction of MICA and MICB on the surface of MCC cells results in increased killing by cytotoxic cells. The circumstance that MCC cell lines grow in spheroids necessitated the use of a flow cytometry based cytotoxic assay. Lymphokine- activated killer (LAK) cells were chosen as effector cells because they are a clinically applicable heterogeneous population of NKG2D^+^, interleukin 2 (IL-2) activated NK, NKT and T cells^32^ and we are currently conducting a clinical trial based on the antibody targeted delivery of IL-2 to the MCC tumor microenvironment (www.immomec.com). The gating strategy to differentiate dead from living cells is illustrated in Fig. 5a for untreated or treated MCC cells (BroLi) at an effector to target ratio of 40:1. In line with the magnitude of the induced membrane expression of MICA/B, the highest LAK cell mediated cytotoxicity was observed for MCC cells treated with the combination of vorinostat and mithramycin A, whereas mithramycin A alone had no and vorinostat alone only an intermediate effect (Fig. 5b). Notably, the LAK cell mediated cytotoxicity correlated with the surface expression of MICA/B. A blocking experiment using saturating amounts of an anti-MICA/B antibody confirmed that increased lysis of MCC cells was indeed dependent on the induction of MICA/B molecules (Fig. 5c). Hence, vorinostat and mithramycin A mediated induction of MICA/B molecules is responsible for the augmented sensitivity of treated MCC cells towards LAK cell mediated lysis.

### MICB expression can be re-induced on MCC cells *in vivo*

**Figure 6:**
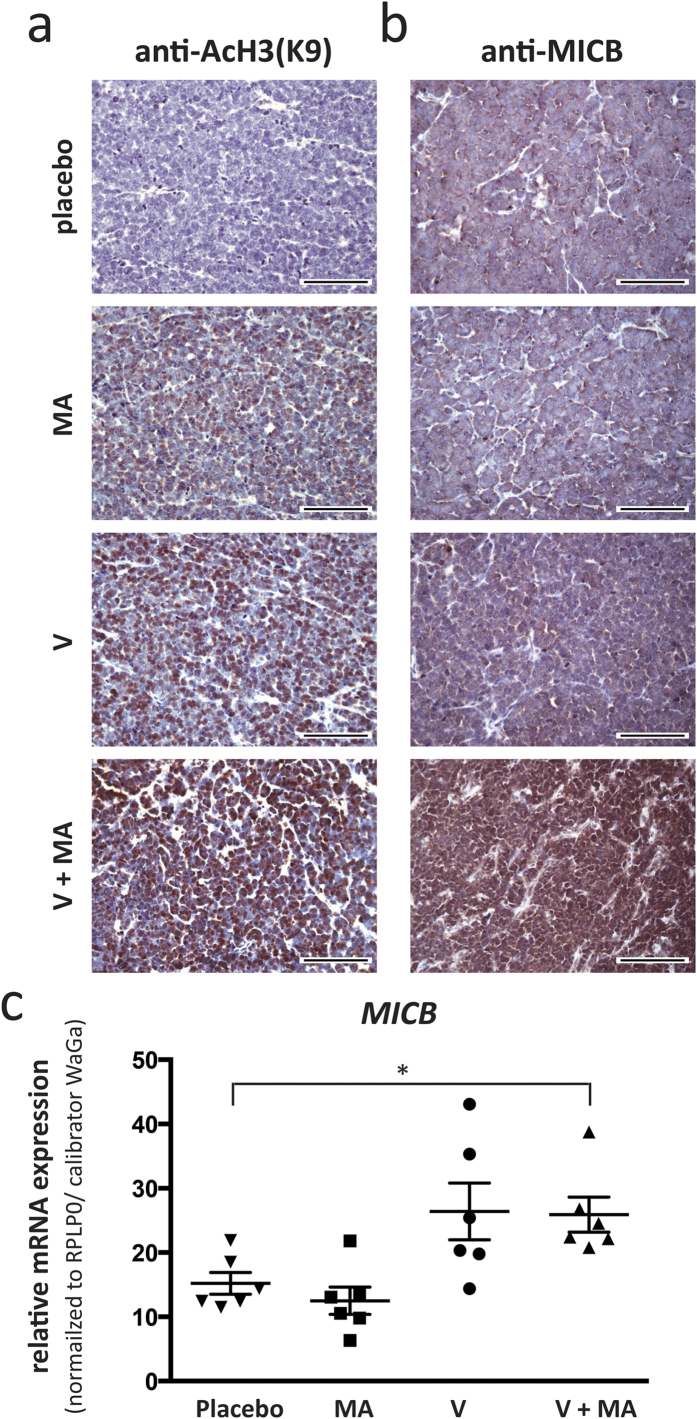
Vorinostat and mithramycin A treatment induces histone H3K9 acetylation and MICB expression in MCC cells *in vivo*. NOD.CB17/Prkdc^scid^ mice (n = 6 for each treatment group) bearing subcutaneous xenotransplants of WaGa cells were treated with placebo, vorinostat (V) mithramycin A (MA), or the combination thereof (V+MA) as described in materials and methods. Immunohistochemistry on FFPE fixed tumor samples obtained after two weeks of treatment was performed using antibodies specific against AcH3K9 (**a**) or MICB (**b**). Representative examples are depicted at 40× magnification, scale bar is 100 μm. (**c**) mRNA was isolated from cryopreserved tumors and qRT-PCR was performed using primers specific for MICB. C_T_ values were normalized to RPLP0 and calibrated to *in vitro* cultured WaGa cells. Statistical analysis was performed using the Kruskal-Wallis test.

To translate these *in vitro* observations into a preclinical *in vivo* setting, we took advantage of a recently established MCC xenotransplantation model in which MCC tumors are induced by subcutaneous injection of WaGa cells in NOD/SCID mice^33^. Once tumors reached a volume of approximately 100 mm^3^, mice were treated for two weeks by intraperitoneal injections of placebo, vorinostat, mithramycin A or the combination thereof at concentrations resulting in serum levels corresponding to those in the clinical setting in humans. After the last dosage animals were sacrificed, and the xenotransplants subjected to detailed characterization. By means of immunohistochemistry, we confirmed both the induction of histone H3 acetylation as well as MICB protein expression (Fig. 6a). In xenotransplanted tumors of mice treated with placebo, histones were hypo-acetylated and inhibition of HDACs induced histone H3K9 acetylation *in vivo*. A substantial induction of histone acetylation, however, was already achieved by vorinostat or mithramycin A alone; still this induced histone acetylation was further enhanced by the combined therapy. The more pronounced effects of the single agents may be due to either the prolonged exposure of either drug in the *in vivo* experiments (14 days with 10 days of drug administration versus 24h) and/or by other mechanisms such as inflammatory responses, interaction of MCC cells with components of the microenvironment or hypoxia. In accordance with the higher level of histone acetylation in MCC xenotransplants, MICB expression was already present in tumors of untreated mice. Still, the induced histone H3K9 acetylation also resulted in an increased expression of MICB protein (Fig. 6b). Quantification of *MICB* mRNA expression clearly demonstrated the synergism of the drug combination (p < 0.05) (Fig. 6c); however, it was not as evident as in the *in vitro* experiments.

## Discussion

Virally associated cancers are characterized by a pronounced immunogenicity. To this end, the higher prevalence of Merkel cell carcinoma (MCC) in immune compromised patients, the high rate of spontaneous regression and the improved prognosis for patients whose tumor is infiltrated by T lymphocytes prompted the discovery of the Merkel cell polyomavirus (MCPyV). It has been shown that most MCC patients mount specific T-cell responses against epitopes derived from proteins encoded by the transforming MCPyV early genes^10^,^11^, which are persistently expressed in MCC lesions in patients and are required for ongoing growth of MCC cell lines^7^,^8^. Despite this immunogenicity, MCC is a very aggressive cancer causing disease-specific death in more than 40% of the patients after primary diagnosis and of almost all patients once distant metastases occur^3^. It should be further noted, that more than 90% of MCCs arise in fully immune competent patients^12^, suggesting tumor specific immune escape mechanisms must be employed. The two main pathways that allow tumors to escape the immune system are loss of immunogenic determinants and the tumor-driven suppression or desensitization of the immune response. Here, we present strong evidence that epigenetic silencing of the stress induced non-classical MHC class I molecules MICA and MICB is one of the major immune escape mechanisms of MCC. These molecules are ligands of the immune regulatory receptor NKG2D expressed on a variety of cytotoxic effector cells of both the innate and adaptive immune system and, most importantly, the NKG2D-NKG2D ligand system has been identified as being essential for the immune surveillance of cancer^41^.

MICA and MICB are not or only slightly expressed by MCC tumors *in situ* and completely absent on MCC cell lines *in vitro*. We demonstrate that the lack of *MICA* and *MICB* mRNA and protein expression in MCC is largely due to epigenetic silencing via histone hypo-acetylation in their promoter region. This epigenetic silencing is very robust even in the presence of several well-established stress factors known to induce their expression. However, this silencing can be abrogated by treatment with HDAC inhibitors both *in vitro* and *in vivo*. Since the ultimate goal of our studies was to establish a therapeutic approach for MCC, we used a clinically relevant concentration, i.e. concentrations attained in patients treated with the FDA approved HDAC inhibitor vorinostat (SAHA, Zolinza^TM^)^28^. Although this concentration of vorinostat increased histone acetylation at the *MICA* and *MICB* promoter as well as subsequent mRNA and protein surface expression in MCC cell lines, the effects were not very strong. This outcome was not entirely surprising, given the fact that HDAC inhibitors are very potent treating hematological malignancies, but have so far failed to achieve significant effects as mono-therapy for solid tumors *in vivo*^34^. Classical MCC cell lines grow as 3D-cultures in large spheroids and therefore represent the *in vivo* situation of a solid tumor much closer than other cancer cell lines^35^. To increase the susceptibility of cancers to HDAC inhibitors, they are frequently combined with other drugs^36^. Mithramycin A is a gene selective Sp1 inhibitor, which has been reported to potentiate HDAC inhibitor induced transcriptional activation^37^. To this end, promoter acetylation of MICA and MICB genes and subsequent mRNA and protein expression were markedly enhanced in MCC cells upon this combined treatment. This, however, is not in line with observations in other cell types where Sp1 appears to be necessary for MICA and MICB transcription and thus mithramycin A inhibits their expression^38^,^39^. Treatment of MCC cell lines with vorinostat or mithramycin A markedly reduced (and the combination almost fully eliminated) Sp1 protein expression. This finding was in line with our observation of decreased Sp1 binding at the *MICA* and *MICB* promoter (Supplementary Fig. 9). Thus, we assume that in MCC cells, Sp1 is not necessary (or when in a complex with certain HDAC molecules even inhibitory) for the transcription of MICA and MICB. This altered Sp1 function may be caused by the presence of MCPyV. Likewise, Venkataraman *et al*.^38^ reported that in cytomegalovirus-infected cells, unlike in non-infected cells, MICA and MICB expression was independent of Sp1.

In a small sub-population of MCC cells, induction of MICA/B surface expression by treatment with HDAC inhibitors in combination with mithramycin A was not as pronounced as in the main population (Fig. 4c and Supplementary Fig. 8a). These cells may exploit resistance mechanisms such as increased drug efflux or represent a slow cycling subpopulation. Since we could detect this subpopulation only by flow cytometry analysis for MICA/B surface expression, other mechanisms to counteract MICA and MICB surface expression, e.g. cytoplasmic retention or shedding from the cell surface, may be operative^14^,^20^.

The principal effect of NKG2D signaling is an enhanced cytotoxic activity of lymphocytes towards the NKG2D ligand-expressing cells. In line with this notion, we observed that HDAC inhibitor treatment of MCC cells resulted in an increased susceptibility to LAK cell-mediated killing. Importantly, this increased cytotoxicity was decreased by a MICA/B blocking antibody. However, this blockade only partially diminished LAK cell mediated cytotoxicity, suggesting that besides MICA and MICB, other immunogenic molecules are induced by the combined treatment with vorinostat and mithramycin A. Indeed, HDAC inhibitors are known to induce the expression of other NKG2D ligands such as ULBPs^40^. It should be further noted that LAK cells include several possible effector cells. NKG2D is expressed as an activating or co-activating receptor not only on NK cells and CD8^+^ T cells, but also on γδ T cells which are important for surveillance of virally associated cancers^19^,^41^. In murine models, γδ T cells are strongly protective against polyomavirus induced tumors; this protection critically depends on their activation via NKG2D^42^.

We recently reported that MCPyV-specific CD8^+^ T cells are present in peripheral blood of more than half of MCC patients^10^, and that intra-tumoral infiltration of CD8^+^ lymphocytes is a positive prognostic marker for these patients^15^. Unfortunately, MCPyV-reactive CD8^+^ T cells are not fully functional in most MCC patients^43^. This exhausted phenotype was associated with expression of PD-1 on the MCPyV-reactive T cells; notably, PD-L1 expression has been reported for both MCC cells and myeloid cells infiltrating the tumor microenvironment^10^,^22^. Signaling via NKG2D may prevent the exhaustion of MCPyV-reactive CD8^+^ T cells. In addition to restoring preexisting T-cell responses, induction of NKG2D ligand expression on MCC cells is likely to trigger new T-cell responses. Activation of NK and γδ T cells via NKG2D increases tumor cell killing and thus cross-presentation of antigens, as well as production of chemokines and cytokines attracting and activating CD8^+^ T cells^44^,^45^. Furthermore, naïve CD8^+^ T cells express NKG2D as a co-activating receptor and binding to NKG2D ligands boosts their activation^46^. In preclinical models it is well established that NKG2D ligand over-expression on tumor cells results in an increased priming and activation of tumor-specific CD8^+^ T cells and long lasting T-cell memory responses even against NKG2D-negative tumor cells^47^: (i) Induction of NKG2D ligands on carcinoma cells boosts anti-tumor effects of CTLA-4 blockade^48^, and (ii) treatment with immune stimulating cytokines such as IL-2 and IL-12 is more effective against NKG2D ligand expressing tumors^49^.

Thus, HDAC inhibitor mediated MICA and MICB induction in MCC is likely to enhance the effects of immune therapeutic approaches currently tested in the clinic: (i) autologous MCPyV specific CD8+ T cell transfer (NCT01758458), (ii) CTLA-4 blocking antibody ipilimumab (NCT02196961), (iii) PD-L1 blocking antibody MSB0010718C (NCT02155647), or cytokine based therapies using (iv) tumor-stroma targeting antibody-IL2 fusion proteins (NCT02054884) or (v) IL12-encoding plasmids delivered by electroporation (NCT01440816).

A limitation of our study is the use of allogeneic LAK cells as effector cells in the cytotoxicity experiments. Unfortunately, autologous peripheral blood or tumor infiltrating lymphocytes from the same patients the respective MCC cell lines were derived from, are not available. LAK cells are a heterogeneous population of highly activated T, NK and NKT cells; hence, it was not possible to further scrutinize the detailed mechanisms by which HDAC inhibition in MCC cells boosts their susceptibility to immune recognition. Furthermore, we focused in this study on the transcription and membrane expression of only MICA and MICB. The expression of other NKG2D ligands is likely to be regulated by histone acetylation as well; however, the fact that the increased susceptibility of MCC cells after inhibition of histone acetylation to LAK cell mediated cytotoxicity is abrogated by an MICA/B blocking antibody strongly argues that induced MICA and MICB expression is the dominating effect.

Recently, many exciting developments have led to new, effective cancer immunotherapies^50^. Immune checkpoint blockade, cytokines with and without tumor targeting, as well as adoptive T cell transfer with and without chimeric antigen receptors results in objective, long lasting clinical responses with response rates, speed and depth even in advanced tumor stages^51^. However, a majority of patients still do not benefit from therapy. Predictive biomarkers for response to immunotherapy are immune response gene signatures or the presence of clonally expanded CD8+ T cells within the tumor^52^. Unfortunately, only 20% of the patients’ MCC lesions are characterized by such a favorable immune signature^15^. The lack of MICA and MICB expression of MCC cells is likely to contribute to this immunological state as re-induction of these NKG2D ligands by HDAC inhibition restores the susceptibility of MCC cells to cytotoxic lymphocytes. Thus, “epigenetic priming” of cancer cells for immune recognition appears to be a valuable addition to current immune therapeutic interventions for MCC^53^.

## Material and Methods

### Patients

A total of 50 archived paraffin-embedded tumor samples from 34 MCC patients were selected from the Department of Dermatology, Medical University of Graz. All tumor samples were histologically confirmed MCC lesions, i.e. primary tumors, local recurrences as well as skin and nodal metastasis. Utilization of the tumor specimens for this study was approved by the institutional review board of the Medical University of Graz (24-295 ex 11/12) and the methods were carried out in accordance with the approved guidelines.

### Immunohistochemistry (IHC)

IHC was performed on formalin fixed and paraffin embedded (FFPE) tissue using the Autostainer Link 48 (Dako, Glostrup, Denmark). After deparaffinization in xylene, sections were rehydrated with 100%, 96%, 70%, and 50% ethanol for 5 min each and finally rinsed with demineralized water. Antigen retrieval for the anti-MICA antibody was performed with EDTA (1mM EDTA, 0.05% Tween 20, pH 8.0) and for the anti-MICB antibody with citrate (Dako retrival solution, cat. no. S1699, pH 6) in a steamer at 100 °C for 30 minutes. After cooling for 20 minutes and two additional washing steps, sections were blocked with peroxidase blocking solution (Dako) followed by incubation over night at 4 °C with antibodies to MICA (AF130, R&D Systems, MN, USA) or MICB (orb 1241, Biorbyte, Cambridge, UK) diluted in antibody diluent (Dako) to 1:200 or 1:100, respectively. After washing steps, incubation with a biotinylated secondary antibody, further washing steps, addition of streptavidin peroxidase, detection was obtained using ImmPACT NovaRED Peroxidase Substrate (Vector Laboratories, Burlingame, CA, USA) according to the manufacturer’s instructions. After counterstaining of nuclei with haematoxylin (Dako), sections were dehydrated and mounted in Tissue Tek glass mounting medium (Sakura Finetek, Torrance, CA, USA). Three independent investigators (CR, DS, JCB) classified the tumors as positive or negative for expression of MICA and MICB.

### Cell culture

The MCC cell lines WaGa, MKL-1, MKL-2, BroLi, AlDo, LoKe^54^,^55^ and melanoma cell lines FM79, FM82, IF6^56^ have been described before. All cell lines were maintained in RPMI-1640 (PAN Biotech, Aidenbach, Germany) supplemented with 10% fetal bovine serum (Sigma, St. Louis, MO, USA) and 1% penicillin/streptomycin (Biochrome, Berlin, Germany). For the cell line AlDo the medium was additionally supplemented with 30% fibroblast conditioned medium. For treatment with specific inhibitors, cells were cultured at a concentration of 1 × 10^6 ^cells/ml in 6 well plates. Inhibitors were dissolved according to the manufacturers’ guidelines and used at 1.25 μM vorinostat (Selleckchem, Munich, Germany), 0.3 μM mithramycin A (Sigma) and 0.3 μM trichostatin A (Selleckchem) for 24 hours if not otherwise stated.

### Quantitative real time-PCR

RNA of *in vitro* propagated cells or cryopreserved xenotransplants was isolated using PeqGOLD total RNA Kit (Peqlab, Erlangen, Germany) and transcribed into cDNA with the Transcriptor First Strand cDNA Synthesis Kit (Roche Life Science, Indianapolis, IN, USA) according to the manufacturer’s instructions. Quantitative real time polymerase chain reactions (qRT-PCR) were performed using SYBR green or TaqMan PCR master mix (Sigma) on the StepOnePlus Real-Time PCR system (Applied Biosystems, Foster City, CA, USA). *RPLP0* was used as endogenous control, and detected with the sense-primer: CCA TCA GCA CCA CAG CCT TA, the antisense-primer: GGC GAC CTG GAA GTC CAA CT, and the probe ATC TGC TGC ATC TGC TTG GAG CCC A. *MICA* and *MICB* mRNA was detected using the SYBR green primers: MICA/B-sense: CAC CTG CTA CAT GGA ACA CAG C, MICA-antisense: TAT GGA AAG TCT GTC CGT TGA CTC T, and MICB-antisense: ACA TGG AAT GTC TGC CAA TGA TC. Relative quantification was calculated by the ΔΔC_T_ method using the melanoma cell line IF6 as calibrator.

### Immunoblotting

Cell lysates were generated by lysing 3 × 10^6 ^cells per sample in protein extraction buffer supplemented with a proteinase inhibitor cocktail as described before^57^. Lysates were subjected to sodium dodecyl sulfate polyacrylamide gel electrophoresis (SDS-Page), samples were transferred to a nitrocellulose membrane (Bio-Rad, Hercules, CA, USA), blocked for 1 hour in a blocking buffer according to the respective antibody’s data sheet and then incubated overnight at 4 °C with primary antibodies diluted in phosphate buffered saline (PBS) with 0.1% Tween 20 (PBST) or tris buffered saline with 0.1% Tween-20 (TBST) according to data sheet: MICB (R&D Systems, Minneapolis, MN, USA) 1:1000 in PBST, acetyl-histone H3 (Lys9) (Cell Signaling Technology, Danvers, MA, USA) 1:1000 in TBST, Sp1 (Cell Signaling) 1:1000 in TBST or β-tubulin (Sigma) 1:1000 in PBST. After membranes were washed trice for 10 minutes each in the respective buffer, they were incubated for 1h with the appropriate peroxidase-coupled secondary antibodies (Dako), followed visualization using the ECL Western Blotting Substrate (Pierce, Rockford, IL, USA).

### Flow Cytometry

Cell surface expression of MICA and MICB was determined by flow cytometry. 1 × 10^6 ^cells were washed with ice cold PBS and incubated with the PE-conjugated anti-human MICA/B antibody (6D4; Biolegend, San Diego, CA, USA) in PBS with 0.1% bovine serum albumin (BSA) for 90 minutes at 4 °C in the dark. After washing steps, cells were stained with 10 μg/ml 7-aminoactinomycin (7AAD, Sigma) to exclude non-viable and measured on a FC500 Flow Cytometer (Beckman Coulter, Brea, CA, USA). Flow cytometry data were analyzed with FlowJo Version 8.7 software (TreeStar, Sunnyvale, CA, USA).

### Chromatin immune precipitation (ChIP)

ChIP assays were performed using the SimpleChiP® Enzymatic Chromatin IP Kit with Agarose beads (Cell Signaling). In brief, proteins were cross-linked to DNA with 1.5% formaldehyde for 10 minutes. Nuclear membranes were broken up using the UP50H Sonicator (Hielscher, Teltow, Germany) set to 100%, 0.9 output for 20 seconds, 6 times in a row with incubation on ice for 30 seconds between sonication pulses. Afterwards, antibodies against histone H3 (cat. no. 6420), acetyl-histone H3 (Lys9) (AcH3K9) (cat. no. 9671), or normal rabbit IgG (Cell Signaling) were used for immune precipitation. The immune precipitated DNA was subsequently analyzed by qRT-PCR, using SYBR green primers specific to the *MICA* or *MICB* promoter region: *MICA* promoter sense CGG ATC CTG GAA TAC GTG GG, antisense ACT CAC ACC TGC CCG TTA TG; *MICB* promoter sense GCG ACA GGG TCC AGG TCG TGC TC, antisense CCC TAC GTC GCC ACC TTC TCA GCT. The percentage of acetylated histones (AcH3K9) was normalized to total Histones H3 and calculated using following equation:


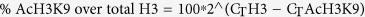


### Flow cytometry based cytotoxicity assay

Peripheral blood mononuclear cells (PBMCs) where isolated via gradient centrifugation with Lymphoprep^TM^ (Stemcell Technologies, Vancouver, BC, Canada), and cultured for 3 days in CellGro®SCGM (CellGenix, Freiburg, Germany) supplemented with 10% FBS (Invitrogen, Grand Island, NY, USA) and 500 IU interleukin 2 (IL-2) per ml (Miltenyi Biotec, Bergisch Gladbach, Germany) to generate lymphokine-activated killer (LAK) cells. MCC cell lines served as target cells either without or with inhibitor (Vorinostat, mithramycin A, or combination thereof) treatment for 12 hours at the concentrations described above; the shortened incubation time was chosen to assure that cells are indeed vital and target cell membranes are fully intact for the cytotoxicity assay. After target cells were washed 3 times in RPMI with 10% FBS, they were labeled by incubation in 3 μM CFSE (Sigma) in pre-warmed RPMI with 10% FBS for 10 minutes at 37 °C, followed by another round of 3 washing steps to remove any excessive CFSE.

2 × 10^4^ CFSE labeled target cells were incubated alone to establish spontaneous cell death, or co-incubated at varying effector:target ratios, i.e. 40:1, 20:1, 10:1, for 4h at 37  °C in a total volume of 100 μl. Before flow cytometry, cells incubated in 10 μg/ml 7AAD (Sigma). Dead target cells were defined as CFSE^+^/7AAD^+^, and the percentage of cytotoxicity was calculated as following:


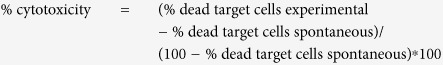


For blocking experiments target cells were incubated with saturating concentrations of blocking antibodies against MICA/B (clone 6D4; Biolegend) or isotype control for 2h at 37 °C. before incubation with LAK cells. Pre incubation with F(ab’)2 fragments for 30 minutes (Life Technologies) was performed to avoid Fc-receptor mediated antibody-dependent cell-mediated cytotoxicity.

### Xenotransplantation experiments

Six-week-old female NOD.CB17/*Prkdc*^scid^ mice were obtained from Charles River Laboratories, and housed under specific pathogen-free conditions. Tumors were induced by s.c. injection as described before^30^. Twenty-four days after tumor cell inoculation, when the tumors reached a volume of approximately 100 mm^3^, treatment was started. Mice were divided into four groups of six mice each ensuring an equal overall tumor burden. Immediately before injection a 1 M (264.3 mg/ml) vorinostat stock solution was diluted in 45% Polyethylene glycol (PEG-400, Sigma) to 10 mg/ml and 60 mg/kg bodyweight were administered i.p. per mouse. For mithramycin A, a 5 mg/ml stock solution was diluted in H_2_O to 0.33 mg/ml and 0.2 mg/kg bodyweight were injected i.p. per mouse. The placebo group received the same volume of the respective solvents. Mice were treated five consecutive days a week for two weeks. Afterwards tumor tissue was formalin fixed and paraffin embedded for IHC or cryo-preserved for RNA isolation. All animal studies had been approved by the Austrian ministry of education and science according to the regulations for animal experimentation (BMWF-66.010/0151-II/3b/2012).

### Statistical Analyses

Statistical analyses were performed using Graphpad Prism 6.0 Software (Graphpad Software Inc., San Diego, CA, USA). Cell culture experiments were analyzed using Friedman test, a paired non-parametric ANOVA. The xenotransplantation experiments were analyzed using Kruskal-Wallis test, an unpaired non-parametric ANOVA. A p-value smaller than 0.05 was considered significant; the respective p-values are indicated in the figures as follows: *p < 0.05; **p < 0.01; ***p < 0.001.

## Additional Information

**How to cite this article**: Ritter, C. *et al*. Reversal of epigenetic silencing of MHC class I chain-related protein A and B improves immune recognition of Merkel cell carcinoma. *Sci. Rep.* **6**, 21678; doi: 10.1038/srep21678 (2016).

## Supplementary Material

Supplementary Information
