# Supplementary material for: Reversal of epigenetic silencing of MHC class I chain-related protein A and B improves immune recognition of Merkel cell carcinoma
Source: Sci Rep. 2016 Feb 23;6:21678. doi: 10.1038/srep21678 (PMC4763224; doi:10.1038/srep21678)
Supplement: Supplementary Information [file srep21678-s1.pdf]

Supplementary data to:

Reversal of epigenetic silencing of MHC class I chain-related protein A and B improves immune recognition of Merkel cell carcinoma

Cathrin Ritter[1,2], Kaiji Fan[3], Kelly G. Paulson[4], Paul Nghiem[4], David Schrama[5*], Jürgen C. Becker[1,2*]

[1]Translational Skin Cancer Research, German Cancer Consortium (DKTK), University Hospital Essen, Germany

[2]German Cancer Research Center (DKFZ), Heidelberg, Germany

[3]Department of Dermatology, Medical University of Graz, Graz, Austria

[4]Division of Dermatology and Medical Oncology, Department of Internal Medicine, University of Washington, Seattle, WA

[5]Department of Dermatology, University Hospital Würzburg, Würzburg, Germany

* equal contribution

Supplementary data:

Supplementary methods:

Tissue microarrays

Tumor samples of 134 MCC lesions comprising primary lesions, recurrences, skin and nodal metastasis of 99 patients were included in the tissue microarray (TMA). All patients had MCC, as assessed by two or more pathologists. Diagnoses occurred between the years 1985 and 2011. All materials were obtained from the MCC Data and Tissue Repository at the University of Washington/Fred Hutchinson Cancer Research Center [Seattle, WA; Institutional Review Board (IRB) approval #6585]. Tissue cores of 0.6 mm in diameter were taken in triplicates from each FFPE tumor sample. Specimens were categorized into MICA/B positive or negative in a blinded fashion.

Immunohistochemistry of tissue microarrays

Sections were deparaffinized in xylene, rehydrated with 100%, 96%, 70%, and 50% ethanol and rinsed with demineralized water. After citrate antigen retrieval, samples were blocked with peroxidase blocking solution (Dako). Samples then were incubated for 60 minutes at room temperature with an antibody recognizing both MICA and MICB (clone 6D4) diluted 1:20 in antibody diluent (Dako). After incubation with a biotinylated secondary antibody and streptavidin peroxidase, detection was carried out with Vector NovaRED Peroxidase Substrate (Vector Laboratories) according to the manufacturer's instructions. Nuclei were counterstained with hematoxylin (Dako). Slides were dehydrated and mounted in Shandon Hypermount (Thermo Scientific) glass mounting medium.

Chromatin immunoprecipitation of Sp1 at the *MICA* and *MICB* promoter

Chromatin immunoprecipitation was performed as described in material and methods before but here with an SP1 specific antibody (cat. no. 5931, Cell Signaling) for untreated and vorinostat plus mithramycin A treated cells. Enrichment of Sp1 at the *MICA* and *MICB* promoter was calculated after normalization to background and relative to input using the following equation:

enrichment to input= 100*2^(adjusted input-C_T IP)

Supplementary Tab. 1: HDAC 1-10 qRT-PCR primer

	forward	reverse
**HDAC1**	CACGGACCGGGTCATGACTGT	CTTGCCTTTGCCAGCCCCGA
**HDAC2**	TCAAGGAGGCGGCAAAAA	TGCGGATTCTATGAGGCTTCA
**HDAC3**	CTGTGTAACGCGAGCAGAAC	GCAAGGCTTCACCAAGAGTC
**HDAC4**	CTGGTCTCGGCCAGAAAGT	CGTGGAAATTTTGAGCCATT
**HDAC5**	CCATTGGAGACGTGGAGTACCT	GCGGAGACTAGGACCACATCA
**HDAC6**	GGAATGGCATGGCCATCATTAG	CGTGGTTGAACATGCAATAGC
**HDAC7**	CTCAGTGGCCATCGCCTGCC	TTGCTGGGTGCCGTTGCCAT
**HDAC8**	GACCGTGTCCCTGCACAAA	CAACATCAGACACGTCACCTGTT
**HDAC9**	CTTCTCACGGACAACAGGGT	GCTCAGCAAAGAATGCACAG
**HDAC10**	ATGTTGCAGTGCCATCCT	GTGTAAATGCTCCCACCTTG

Legends to the supplementary figures:

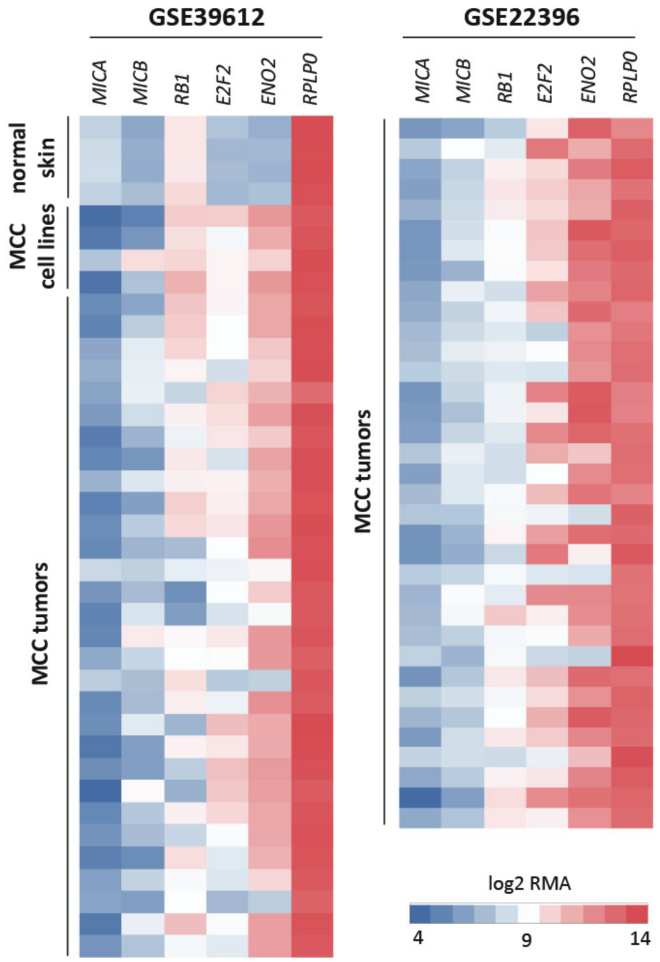

Supplementary Fig. 1: *MICA* and *MICB* mRNA expression in MCC lesions *in situ*.

Robust multi array average (RMA) normalized expression values of two gene expression arrays (GSE22396 and GSE39612) were obtained from the Gene Expression Omnibus (GEO) database. In GSE 22396 the gene expression profiles of 35 MCC tumors of 34 patients were analyzed. In GSE39612 data of 30 MCC tumors of 27 patients and 4 MCC cell lines renormalized with normal skin samples were provided. RMA values were log2 transformed and are depicted as heat map with expression values ranging from 4 (blue = low expression) to 14 (red = high expression). *MICA* and *MICB* mRNA expression is shown in comparison to *RB1*, *E2F2*, *ENO2* and *RPLP0*.

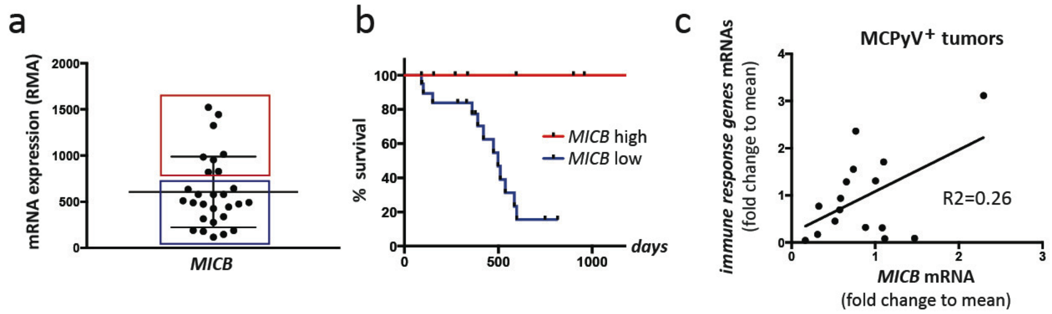

Supplementary Fig. 2: High *MICB* expression in MCC tumors is associated with an improved survival and immune infiltration.

Robust multi array average (RMA) normalized expression values of *MICB* mRNA were obtained from the Gene Expression Omnibus (GEO) database (GSE22396). **(a)** Tumors were categorized according their MICB mRNA expression level: The low group persists of tumors with a *MICB* mRNA expression below or equal to average (within blue square), the high group includes tumors with a *MICB* mRNA expression markedly above average (within red square). **(b)** MCC-specific survival was analyzed using the Kaplan-Meier method for the *MICB* low (blue line) or high (red line) mRNA expressing tumors; p=0.009 (c) *MICB* mRNA expression was correlated with the expression of immune gene signature associated with a good prognosis (i.e. *ALDH1A, AMICA1, BHLHE41, CCL 19, CCR2, CD8a, CGA, CHI3L1, CHIT1, CHRNA9, FAM46C, FBP1, GZMA, GZMB, GZMH, GZMK, HLA-DPA1, HLA-DRB5, IGJ, IGKC, ITGBL1, KLRK1, LYZ, MMP7, POUF2AF1, PROM1, SLAMF1, TRBC1)* for MCPyV positive tumors by linear regression analysis (p=0.04 , R2=0.26).Tumors were classified into MCPyV positive and negative by their *MCPyV T-Antigen* mRNA expression state.

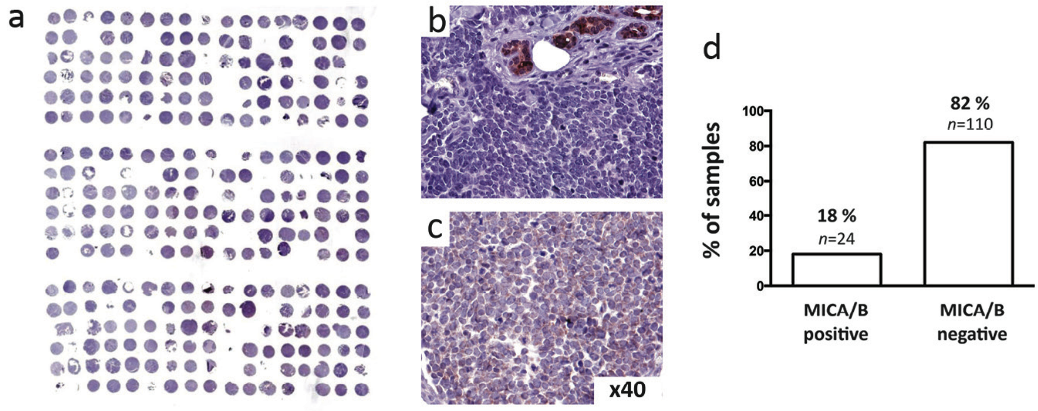

Supplementary Fig. 3: ***in situ*** **expression of MICA/B in MCC lesions in tissue microarrays.** Triplicates of 134 MCC tumors of 99 patients were arranged on a tissue microarray as shown **(a)**. MICA/B expression was determined by immunohistochemistry using an antibody recognizing both MICA and MICB (clone 6D4) as described in supplementary material and methods. Samples were classified as positive or negative and representative sections for negative **(b)** and positive **(c)** lesions are depicted at 40x magnification. Percent of MICA/B positivity is given on a lesion based calculation **(d)**.

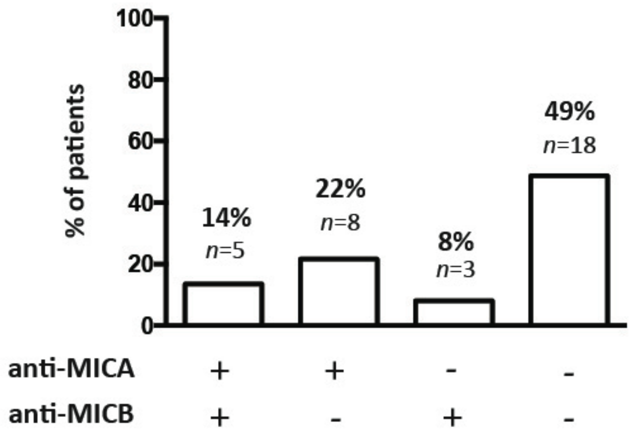

Supplementary Fig. 4: Frequency of *in situ* expression of MICA and MICB in sections of MCC lesions calculated on a per patient basis.

50 MCC tumor samples of 34 patients were analyzed by immunohistochemistry for expression of MICA and MICB. The intra-individual heterogeneity of the analyzed tumor lesions is negligible. Patients were classified into 4 groups: Double positive (+/+, 14%, *n=5*) or double negative (-/-, 49%, *n=18*) for the expression of MICA and MICB, or only positive for MICA (+/-, 22%, *n=8*) or MICB (-/+, 8%, *n=3*). Groups are depicted as percentage of all patients (*n=34*).

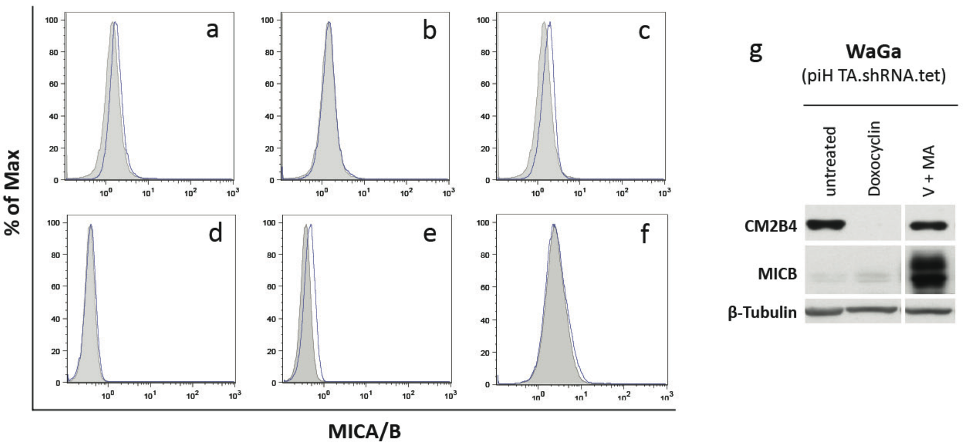

Supplementary Fig. 5: Cell stress does not induce MICA/B protein expression in a MCC cell line.

MICA/B cell surface expression of WaGa cells under standard culture conditions (grey filled area) or after **(a)** serum starvation, **(b)** 5% DMSO, **(c)** heat shock at 41,5°C for 24hrs, the presence of 1000U/ml **(d)** interferon α or **(e)** interferon γ for 48h, or **(f)** after silencing of the MCPyV-encoded T antigens as illustrated under **(g)** was determined by flow cytometry using an antibody recognizing both MICA and MICB (clone 6D4). A previously described inducible MCPyV T-antigen knockdown was used to silence both MCPyV large and small T-antigens[54]. WaGa (piH TA.shRNA.tet) cells were cultured for 5 days in the absence or presence of 1 µg/ml doxycycline; silencing of LTA was confirmed by immunoblot using the LTA specific antibody CM2B4. MICB protein expression of whole MCC cell lysates was determined by immunoblot; β-tubulin served as a loading control; WaGa (piH TA.shRNA.tet) cells treated with the combination of vorinostat and mithramycin A served as positive control.

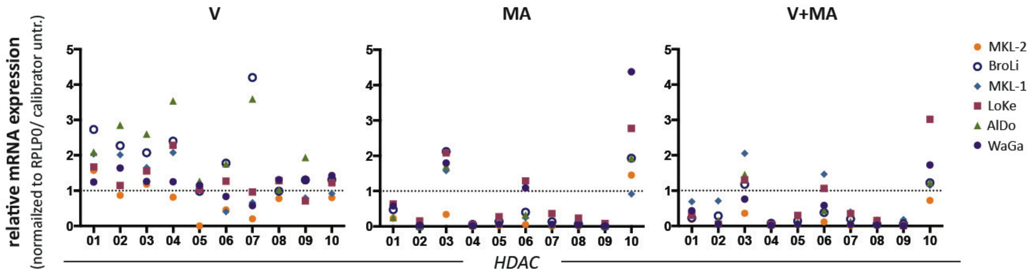

Supplementary Fig. 6: Mithramycin A inhibits vorinostat induced *HDAC* mRNA transcription.

RNA was isolated from the indicated MCC cell lines (MKL-2 [yellow dot], BroLi [blue circle], MKL-1 [blue diamond], LoKe [pink square], AlDo [green triangle], and WaGa [purple dot]) either untreated or treated with vorinostat (V), mithramycin A (MA), or the combination thereof (V+MA) treated as described in Material and Methods. qRT-PCR was performed using primers specific for class I and II *HDACs 1-10* listed in supplementary Table 1. C_T values were normalized to *RPLP0*. The relative mRNA expression compared to the respective untreated cell lines is depicted.

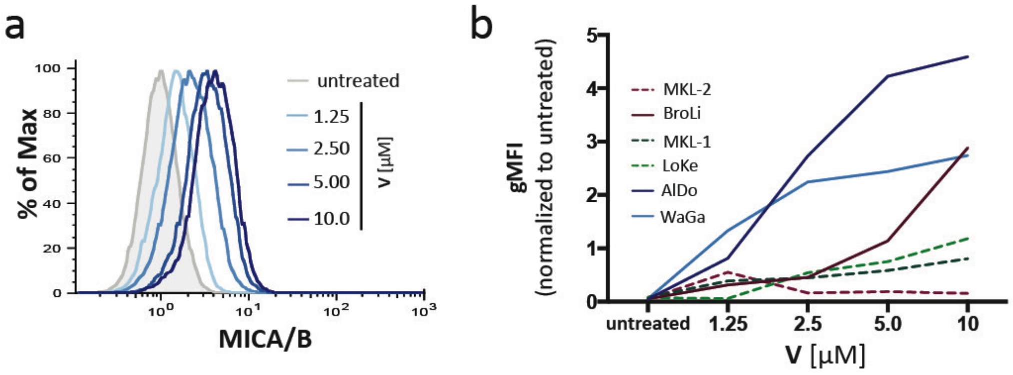

Supplementary Fig. 7: Induction of MICA/B surface expression by vorinostat is dose dependent.

MICA/B cell surface expression MCC cell lines (MKL-2 [purple dashed line], BroLi [purple line], MKL-1 [dark green dashed line], LoKe [light green dashed line], AlDo [dark blue line], and WaGa [light blue line]) cells subjected to increasing concentrations of vorinostat (V)(none, 1.25, 2.50, 5.00 and 10.00 µM) for 24h was determined by flow cytometry using an antibody recognizing both MICA and MICB (clone 6D4), which is exemplified for AlDo **(a)**. The summary for all cell lines is depicted as the geometric mean fluorescence intensity (gMFI) normalized to the respective untreated cell lines **(b).**

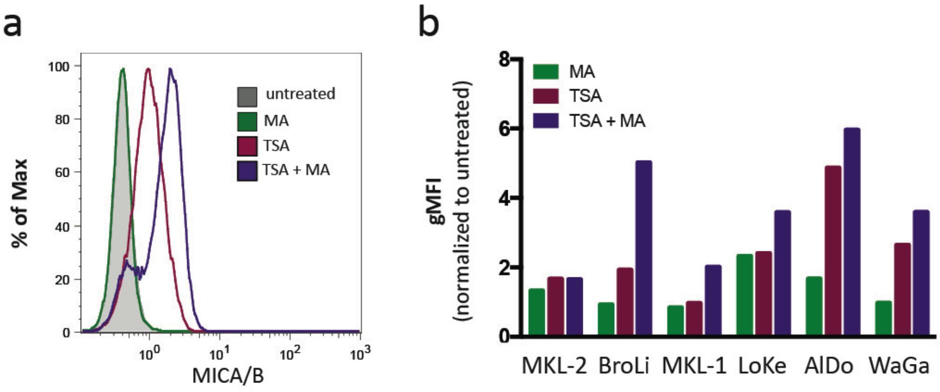

Supplementary Fig. 8: Induction of MICA/B expression by trichostatin A alone or in combination with mithramycin A.

Cell surface MICA/B expression on untreated MCC cell lines (MKL-2, MKL-1, BroLi, and WaGa) was compared to the expression after treatment with mithramycin A (MA, green), trichostatin A (TSA, red), or the combination thereof (TSA+MA, purple) was determined by flow cytometry using an antibody recognizing both MICA and MICB (clone 6D4), which is exemplified for WaGa **(a)**. The data for all cell lines is depicted as the geometric mean fluorescence intensity (gMFI) of MICA/B staining, normalized to the respective untreated cell line **(b).**

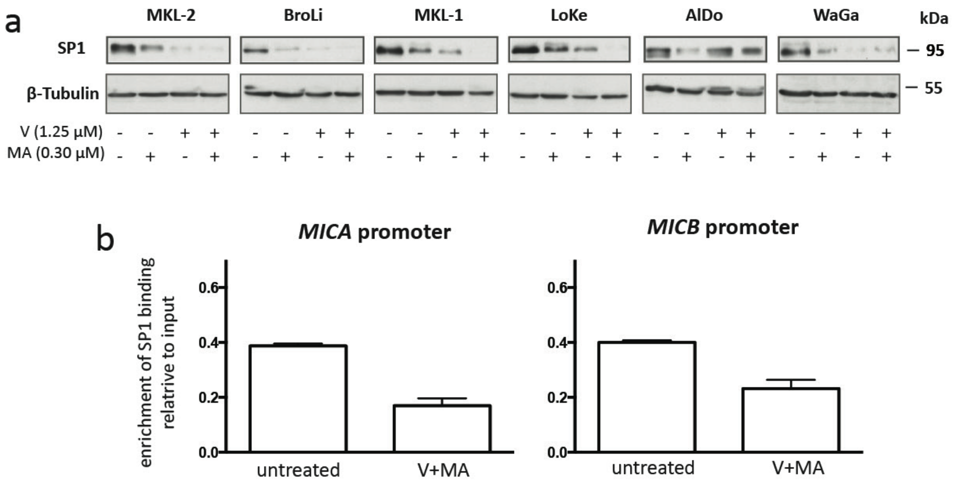

Supplementary Fig. 9: Reduced SP1 expression and *MICA* and *MICB* promoter binding after vorinostat and mithramycin A treatment.

(a) Sp1 expression in whole cell lysates of either untreated or treated with vorinostat (V), mithramycin A (MA), or the combination thereof (V+MA) MCC cell lines was detected by immunoblot using a Sp1 specific antibody; β-tubulin served as loading control. **(b)** Chromatin immunoprecipitation (ChIP) assay was performed with untreated and vorinostat plus mithramycin A (V+MA) treated WaGa cells followed by a qRT-PCR using MICA or MICB promoter specific primers. Sp1 enrichment at the MICA and MICB promoter relative to input was calculated as described in supplementary material and methods. qRT-PCR was performed in duplicates and results are expressed as mean ± SEM
